# A critical analysis of 33 patients with substernal goiter surgically treated by neck incision

**DOI:** 10.1016/S1808-8694(15)30774-6

**Published:** 2015-10-19

**Authors:** Murilo Catafesta Das Neves, Marcello Rosano, Flávio Carneiro Hojaij, Márcio Abrahão, Onivaldo Cervantes, Danielle Macellaro Andreoni

**Affiliations:** 1 MD, Surgeon, Assistant Physician at the Head and Neck Surgery Program at UNIFESP; 2 MD, Surgeon, Assistant Physician at the Head and Neck Surgery Program at UNIFESP; 3 Professor in the Head and Neck Surgery Program at UNIFESP; 4 Professor, Head of the Head and Neck Surgery Program at UNIFESP; 5 Professor, Head of the Head and Neck Surgery Program at UNIFESP; 6 MSc in Endocrinology, Assistant Physician at the Endocrinology Program at UNIFESP. Head and Neck Surgery Program at Universidade Federal de São Paulo

**Keywords:** substernal goiter, surgery, therapy

## Abstract

The possibility of needing a combined access, with neck and chest incisions makes the treatment of substernal goiter a challenge both in the pre-op and the intraoperative. We hereby, discuss a standardization of the surgical technique to minimize the need for a chest approach, making the substernal goiter a surgically treatable disease, through a single neck incision, and with low indices of complication.

**Aim:**

to assess the substernal goiter surgically approach through a neck incision and to analyze the surgical complications.

**Materials and methods:**

we carried out a historical cohort by retrospective analysis of the charts of patients submitted to thyroidectomy, and 33 of them (10.4%) had substernal goiter.

**Results:**

all 33 patients were surgically treated through a neck incision without the need for sternotomy. We did not observe definitive lesions in the inferior laryngeal nerve or definitive hypoparathyroidism. Only 2 patients had recurrent nerve paresis; and 2 patients were re-operated because of a neck hematoma.

**Conclusion:**

patients with substernal goiter can be safely treated surgically through a single neck incision, bearing low complication rates.

## INTRODUCTION

Haller was the first to describe substernal goiter in 1749 as the extension of thyroid tissue below the upper opening of the chest^1^^,^^2^. Today, substernal goiter is characterized when more than 50% of the gland is extended into the chest, thus requiring dissection of the upper mediastinum.^2^

Variations on the definition include thyroid extensions greater than 3cm below the furculum or extensions below the fourth thoracic vertebra, however with no impact on disease classification or prevalence rates.

A preoperative method to determine the need to perform sternotomy in substernal goiter patients is not established. Therefore, cases in which higher morbidity procedures may be required are seen with doubt and insecurity by both physicians and patients. One should mention that some patients suffering from substernal goiter have no symptoms and the mere hint of a sternotomy makes the procedure even less accepted.

Besides, although thyroidectomy is an established procedure with low complication rates, the same cannot be said of substernal goiter^3^, ^4^, ^5^, ^6^, ^7^, ^8^, ^9^, ^10^, ^11^, ^12^, ^13^, ^14^, ^15^.

The treatment for substernal goiter is eminently surgical. This paper aims to confirm the standard surgical technique and analyze its complications, comparing it against data found in the literature.

## MATERIALS AND METHOD

Between May of 2002 and July of 2007, 316 patients underwent surgical treatment for goiter at our institution. Thyroidectomy for substernal goiter was performed in 33 (10%) of them.

Patient mean age was 51 years (ages ranged between 32 and 83 years). Twenty-five were women and 7 were men, a ratio of 3.5:1.

Reported clinical findings were as follows: 10 (30%) patients had dyspnea, 7 (21%) had dysphagia, 7 (21%) had dyspnea and dysphagia, and only 2 (6%) had hyperthyroidism. Seven (21%) did not report clinical complaints.

Neck and chest CT scans were ordered for all patients suspected for substernal goiter to confirm the diagnosis and plan for surgery. Thyroid extension into the mediastinum and tracheal deviation were seen in all suspected cases.

**Figure 1:**
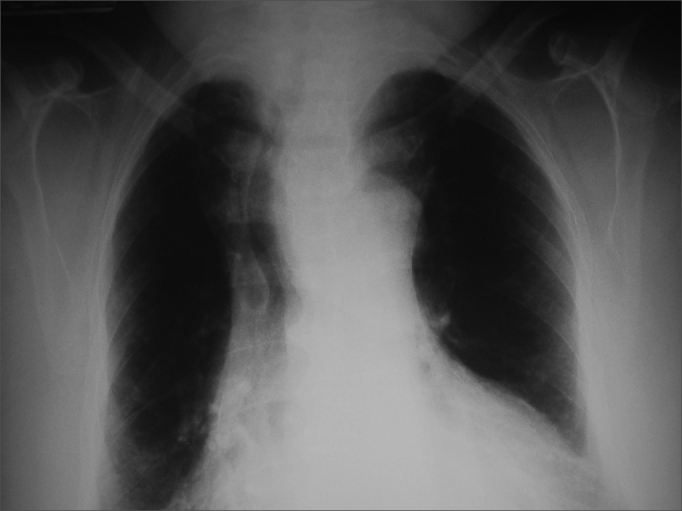
Chest X-ray showing deviated trachea and enlarged upper mediastinum due to substernal goiter.

**Figure 2:**
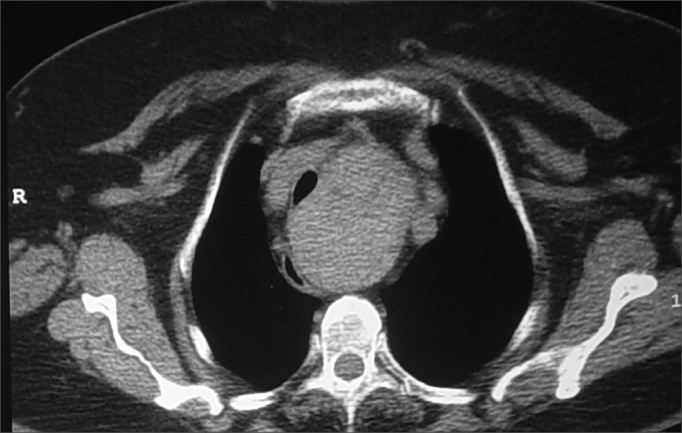
CT scan showing goiter displacing mediastinal structures.

Clinical suspicion is established during physical examination when the lower border of the gland cannot be palpated or found in preliminary images. Additional findings include tracheal deviation and mediastinal mass in chest X-ray images. Figure 1, Figure 2 show respectively X-ray finding in patient suspected for substernal goiter and confirmation through CT scan. Tracheal deviation and/or large size goiter are indicative of the need for surgery.

All patients with preoperative clinical and imaging findings compatible with substernal goiter were included. Patients not meeting the criteria, others diagnosed with substernal goiter intraoperatively, and poor thyroidectomy candidates were excluded. The planned procedure was a neck approach followed by a thyroidectomy. The same surgical procedure was performed in all patients.

This paper was approved by the Research Ethics Committee of our university under permit 0725/05.

### Surgical Technique

**Figure 3:**
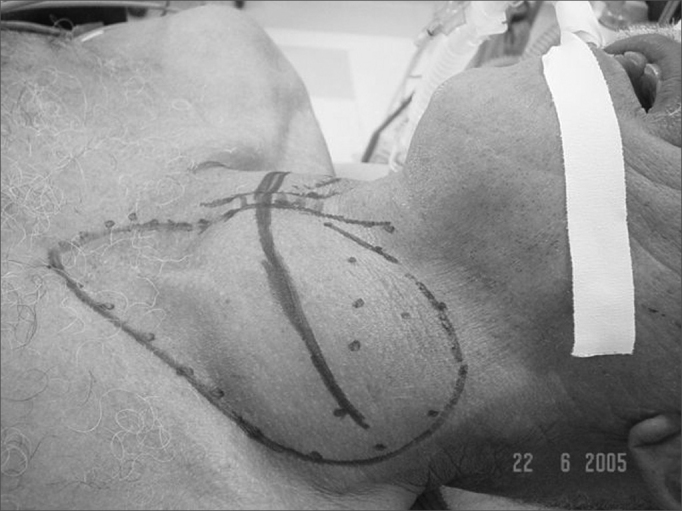
Desenho do bócio com marcação da incisão cirúrgica.

Surgery starts with a broad transverse neck incision to provide good visualization of the structures (Figure 3). Then the platysma muscle is sectioned and cranial and caudal subplatysmal flaps are dissected. The median raphe is opened until the plan of the thyroid.

**Figure 4:**
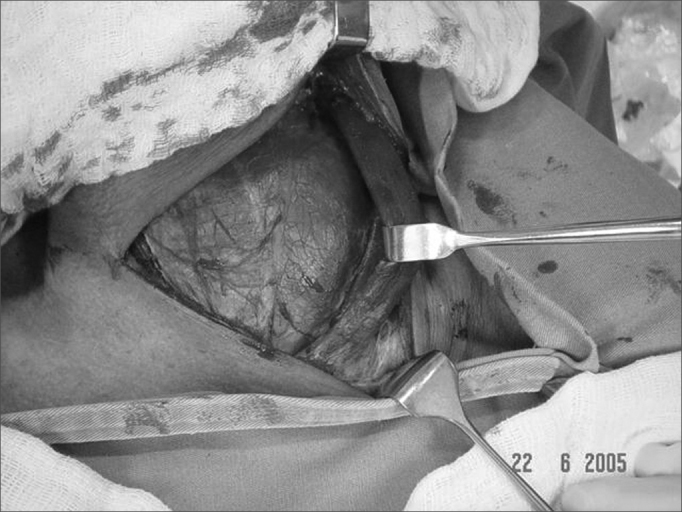
Releasing the sternocleidomastoid muscle during surgery.

**Figure 5:**
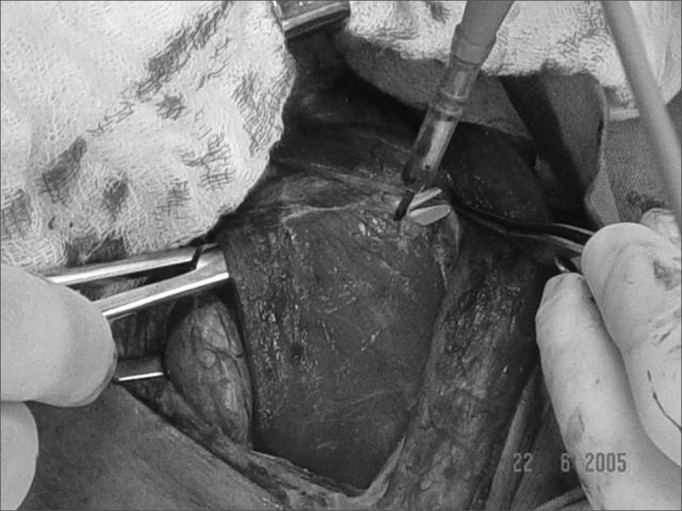
Sectioning prethyroid musculature.

It is important that the procedure is initiated on the side where substernal goiter is larger. Then the sternocleidomastoid muscle (Figure 4) and the prethyroid muscles

(Figure 5) are released, the latter being sectioned in its upper third and retracted laterally, thus enhancing lateral exposure.

**Figure 6:**
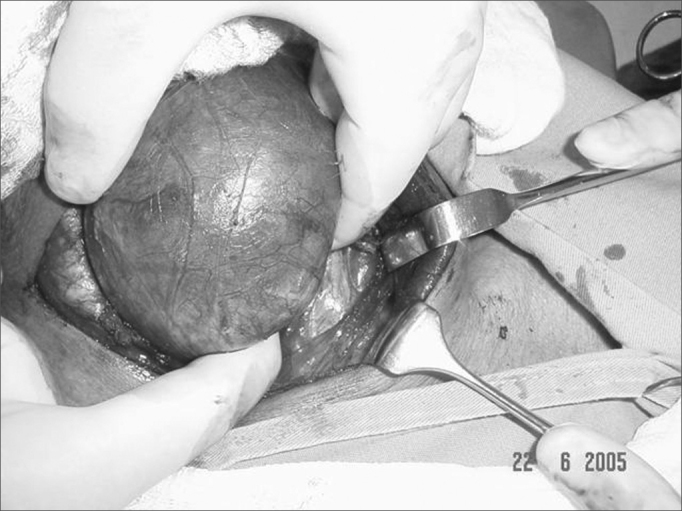
two-finger palpation with dislocation of the substernal goiter lobe.

The upper pole is then ligated through the usual technique and loose tissues adhered to the gland are bluntly dissected, with special attention given to the ones in the upper mediastinum. The diving lobe is then pulled and dislocated (Figure 6). The laryngeal nerve and the parathyroid glands are then identified and spared. The lower pole is ligated and the tracheal gland released.

After the lower substernal lobe is resected the surgical site is usually broad enough not to require the release of the muscles in the contralateral side during the resection of the contralateral lobe.

The procedure is concluded with the usual hemostasis technique, draining using a closed drain tube system, and suture by layers.

## RESULTS

Thirty of the 33 patients underwent total thyroidectomy; all had bilateral multi-nodular goiter; only three were treated with hemithyroidectomy as they had unilateral benign disease. Postoperative pathology tests revealed that 2 (6%) patients had well-differentiated malignant disease, both cases of follicular variant of papillary carcinoma with sizes ranging between 1 and 2 centimeters. These patients had been previously undergone total thyroidectomy. As mentioned above, all patients were submitted to the described surgical technique and none required sternotomy or thoracotomy.

After surgery, the patients stayed at the hospital for an average 3 days (2–7 days). Patients submitted to total thyroidectomy were given two grams of calcium and 0.5 mcg of vitamin D orally per day postoperatively in order to prevent the occurrence of symptoms connected to transitional hypocalcemia observed in about 50% of the patients submitted to total thyroidectomy16. Only two of the patients required medication for over a month after surgery, while none took medication for more than six months into follow-up. Therefore, no cases of permanent hypoparathyroidism were observed.

Only two (6%) patients had lower laryngeal nerve paresis, but the condition subsided two months into follow-up. No cases of permanent lower laryngeal nerve paralysis were recorded. Changes on the outer branch of the upper laryngeal nerve were not objectively assessed, but none of the patients had complaints related to it. Only two (6%) patients were reoperated due to neck hematoma.

## DISCUSSION

Surgery is the treatment of choice for substernal goiter. The procedure can be performed through one neck incision in most cases. Employment of systematic technique minimizes the need for sternotomy, even in patients with significant intra thoracic component, and keeps severe complication rates at levels comparable to those of conventional thyroidectomy.

Prevalence rates of substernal goiter range between 1% and 21% of goiter cases^3^, ^4^, ^5^, ^6^, ^7^, ^8^^,^^17^, ^18^, ^19^; in spite of being a relatively frequent finding, clinical diagnosis is not always obvious.

Patients with enlarged glands can be asymptomatic in 10% to 35% of the cases^3^^,^^4^^,^^6^^,^^7^^,^^9^^,^^10^^,^^17^^,^^19^^,^^20^ while some authors claim such rate to be as high as 50%^8^^,^^9^.

Most patients are diagnosed at a later age, usually above 70 years. They have had goiter for a long time, and only seek medical advice when symptoms kick in. Compressive complaints such as dyspnea are reported by 16–65%^3^^,^^6^, ^7^, ^8^, ^9^^,^^19^^,^^20^ of the patients; dysphagia by 4–27%^3^^,^^6^^,^^9^^,^^19^^,^^20^; and presence of neck mass by 35–100%^3^^,^^7^, ^8^, ^9^, ^10^^,^^20^. Hyperthyroidism is reported in about 13% of the cases^3^^,^^10^.

Alongside clinical suspicion, imaging studies are used to confirm the diagnosis. As far as we see it, all patients clinically suspected for substernal goiter must undergo neck and chest CT scanning, both to confirm the presence of the disease and devise a treatment plan.

The literature is consistent in stating that most substernal goiter cases may be resected through one neck incision. The need for sternotomy or thoracotomy ranges between 0% and 13%^3^, ^4^, ^5^^,^^7^, ^8^, ^9^, ^10^, ^11^, ^12^, ^13^, ^14^^,^^17^^,^^19^; one author reported rates of 29%^7^.

Authors such as Sand et al.^15^ support a broader use of sternotomies so as to avoid excessive traction of mediastinal structures and minimize postoperative complications. Other authors suggest that when more than 70% of the thyroid volume is in the chest, a thoracotomy is inevitable^5^.

Papers^8^^,^^18^^,^^20^ in which higher rates of thoracotomy were presented have however failed to show fewer complications. In reality, regardless of the approach ––– neck or chest ––– complication rates were similar.

Rates of nerve paresis range between 0–7.8%^4^, ^5^, ^6^, ^7^^,^^11^^,^^13^^,^^14^; paralysis occurs in 0–4%^4^, ^5^, ^6^, ^7^^,^^9^^,^^12^^,^^14^^,^^15^ of the cases and permanent hypoparathyroidism in 0–6%^3^^,^^4^^,^^7^, ^8^, ^9^, ^10^, ^11^^,^^13^^,^^14^. All such complications are consistent with the findings observed in our case base; they are, respectively, 6%, 0% and 0%.

Temporary hypoparathyroidism is reported in 1.6–50% (16) of the cases, and may affect as many as 73% of the patients with substernal goiter^21^. Given such high prevalence rate, all patients in our case base were given calcium and vitamin D orally. Hypoparathyroidism, therefore, was not looked into.

Although no sternotomies were required in our case base, we agree with De Perrot et al.^20^ in that such procedure should be reserved to relapsing, ectopic, or neoplasm cases, the latter when there is invasion of mediastinal structures.^20^

Malignancy rates associated with substernal goiter range between 4 and 16%^6^, ^7^, ^8^, ^9^, ^10^^,^^13^^,^^19^^,^^20^. Six percent of the patients in our group were diagnosed with well differentiated tumors. However, diagnosis was obtained only through surgical specimen examination, thus not impacting the approach described in this paper.

## CONCLUSION

Patients with substernal goiter can and should be safely treated through the neck approach with good outcome, while sternotomy should be reserved for selected cases.

## References

[1] Gonçalves J, Kowlski LP (2005). Surgical complications after thyroid surgery performed in a cancer hospital. Otolaryngol Head Neck Surg..

[2] Carvalho MB, Junior CF (2007).

[3] Moran JC, Singer A, Sardi A (1998). Retrosternal Goiter: A six year Institutional Review. The Am Surg..

[4] Siragusa G, Gelarda E, Geraci G. Gozzo cervio-mediastinico. Minerva Chirurgica 544:225-9. 10380520

[5] Flati G, De Giacomo T, Porowska B, Flati D, Gaj F, Talarico C, Antonellis F, Diana M, Berloco PB (2005). Surgical management of substernal goitres. When is sternotomy inevitable?. Clin Ter..

[6] Maruotti RA, Zannini P, Viani MP, Voci C, Pezzuoli G (1991). Surgical treatment of substernal goiters. Int Surg..

[7] Erbil Y, Bozbora A, Barbaros U, Ozarmagan S, Azezli A, Molvalilar S (2004). Surgical management of substernal goiters: clinical experience of 170 cases. Surg Today.

[8] Sciumè C, Geraci G, Pisello F, Li Volsi F, Facella T, Modica G (2005). Substernal goitre. Personal experience. Ann Ital Chir.

[9] Chow TL, Chan TT, Suen DT, Chu DW (2005). Lam SH Surgical management of substernal goitre: local experience. Hong Kong Med J..

[10] Torre G, Borgonovo G, Amato A, Arezzo A, Ansaldo G, De Negri A, Ughè M, Mattioli F (1995). Surgical management of substernal goiter: analysis of 237 patients. Am Surg..

[11] Makeieff M, Marlier F, Khudjadze M, Garrel R, Crampette L, Guerrier B (2000). Substernal goiter. Report of 212 cases. Ann Chir..

[12] Wang LS, Shai SE, Fahn HJ, Chan KH, Chen MS (1994). Huang MS Surgical management of substernalgoiter. Scand J Thorac Cardiovasc Surg.

[13] Ben Nun A, Soudack M, Best LA (2006). Retrosternal thyroid goiter: 15 years experience. Isr Med Assoc J..

[14] Arici C, Dertsiz L, Altunbas H, Demircan A, Emek K (2001). Operative management of substernal goiter: analysis of 52 patients. Int Surg..

[15] Sand ME, Laws HL, McElvein RB (1983). Substernal and intrathoracic goiter. Reconsideration of surgical approach.. Am Surg..

[17] Grainger J, Saranappa N, D'Souza A, Wilcock D (2005). The surgical approach to retrosternal goiters: The role of coputerized tomography. Otolaryngol Head Neck Surg..

[18] Moncick JM, Materazzi G (2000). The Necessity for a Thoracic Approach in Thyroid Surgery. Arch Surg..

[19] Netterville JL, Coleman SC, Smith JC, Smith MM, Day TA, Burkey BB (1998). Management of substernal goiter. Laryngoscope.

[20] de Perrot M, Fadel E, Mercier O, Farhamand P, Fabre D, Mussot S, Dartevelle P (2007). Surgical management of mediastinal goiters: when is a sternotomy required?. Thorac Cardiovasc Surg..

[21] Higgins KM, Mandell DL, Govindaraj S, Genden EM, Mechanick JI, Bergman DA, Diamond EJ, Urken ML (2004). The Role of Intraoperative Rapid Parathyroid Hormone Monitoring for Predicting Thyroidectomy-Related Hypocalcemia. Arch Otolaryngol Head Neck Surg..

